# Brief videoconferencing psychological intervention for reducing COVID-19 related distress: study protocol for a randomized controlled trial

**DOI:** 10.1186/s12889-021-10529-x

**Published:** 2021-03-09

**Authors:** Dharani Keyan, Katie Dawson, Suzanna Azevado, Srishti Yadav, Jenny Tran, Richard A. Bryant

**Affiliations:** School of Psychology, University of New South Wales, Sydney, NSW 2052 Australia

**Keywords:** COVID-19, Controlled trial, Psychosocial intervention, Psychological distress

## Abstract

**Background:**

Globally COVID-19 has had a profound impact on the psychological wellbeing of millions of people, and there is an urgent imperative to address elevated levels of distress during the COVID-19 pandemic. The World Health Organization (WHO) has developed Problem Management Plus (PM+), a low intensity psychological intervention for adults experiencing psychological distress. This paper outlines the study protocol for a trial that tests the effectiveness of an adapted version of PM+ to reduce distress associated with COVID-19.

**Methods:**

A single-blind, parallel, randomized controlled trial will be carried out for distressed people across Australia. via video conferencing on a small group basis. Following informed consent, adults that screen positive for levels of psychological distress (General Health Questionnaire-12 (GHQ-12 score ≥ 3) and have access to videoconferencing platform will be randomised to an adapted version of gPM+ (*n* = 120) or enhanced treatment as usual (ETAU) (n = 120). The primary outcome will be reduction in psychological distress including anxiety and depression at 2-months post treatment. Secondary outcomes include worry, sleep problems, anhedonia, social support, and stress in relation to COVID-19.

**Discussion:**

The trial aims assess whether an adapted version of videoconferencing PM+ that is specifically designed to target COVI-19 related distress will result in reduced distress relative to enhanced usual care.

**Trial registration:**

This trial was prospectively registered on the ANZCTR on 14/4/20 (ACTRN12620000468921).

## Article summary

### Strengths and limitations of the study

- This is the first controlled trial of a videoconferencing psychological intervention to reduce psychological distress during COVID-19.
- This design is novel in that it provides remote delivery and is group-based which affords potentially cost-effective mental health intervention in situations of lockdowns.
- This trial conducts blind assessments of all participants at baseline, post-intervention, 2-month follow-up, and 6-month follow up via online assessments.
- Structured clinical interviews were not conducted to ascertain mental disorders.
- The study does not provide long-term follow-up assessments beyond 6 months, thereby limiting conclusions about longer-term benefits.

## Background

The COVID-19 pandemic, caused by severe acute respiratory syndrome coronavirus 2, has had a profound global impact, and represents the most severe global health threat in recent pandemic history. Concerns about COVID-19 are pervasive and have several serious adverse impacts on hospital systems, health workers and the general community. A pandemic of this nature with the associated uncertainty including lockdowns, physical distancing and other quarantine measures has been shown to involve a significant psychological burden on many people. Many studies conducted during the COVID-19 pandemic have highlighted the elevated rates of common mental disorders, with systematic reviews and meta-analyses of studies conducted throughout the pandemic indicate that between one-quarter to one-third of people in affected communities may be experiencing these conditions [1–4]. For example, one meta-analysis indicated that the pooled prevalence of depression, anxiety, psychological distress, and insomnia was 31.4, 31.9, 41.1, and 37.9%, respectively [2]. Various factors have been shown to increase the risk of psychological problems during the pandemic, including being female, younger, lower socioeconomic status, living in rural areas, in quarantine or lockdown restrictions, infected with the virus [2, 5], There is also much evidence that frontline health workers are also at higher risk for common mental disorders [6–8].

There is an urgent imperative for scalable programs that can address the pervasive psychological distress experienced by people during the COVID-19 pandemic. In the light of need for physical distancing and evolving lockdown and quarantine measures, these programs need to be delivered in an easily accessible format that can accommodate social distancing requirements and that can also achieve considerable reach across communities. One possible strategy is the Problem Management Plus (PM) + that has been developed by the World Health Organisation (WHO) in collaboration with the University of New South Wales (UNSW) [9]. This is a 5-session scalable intervention designed to reduce stress, anxiety and depression in the wake of crisis and adversity. It comprises evidence-based techniques of problem solving, stress management, behavioral activation, and accessing social support [10]. This program is suitable for the current needs during COVID-19 because PM+ strategies are simple to learn, are transdiagnostic and address diverse features of psychological distress, and can be taught in brief format. The intervention was developed to be delivered by a range of trained personnel, including those with no specialist mental health qualifications. PM+ has been evaluated in various setting and shown to reduce mental distress and promote general functioning in those exposed to ongoing adversity, and has been shown to be effective when delivered in individual [11, 12] or group [13] formats.

The goal of the current study is to test the effectiveness of an adapted version of PM+ specifically designed to target COVID-19 related distress and coping in the face of ongoing uncertainty and unpredictability of the current situation. We predict that PM+ would lead to greater reductions in anxiety and depression relative to enhanced usual care, as well as reduced worry, sleep disturbance, positive and negative mood, hedonia, and COVID-19 related concerns.

## Methods

### Aim and design

**Fig. 1:**
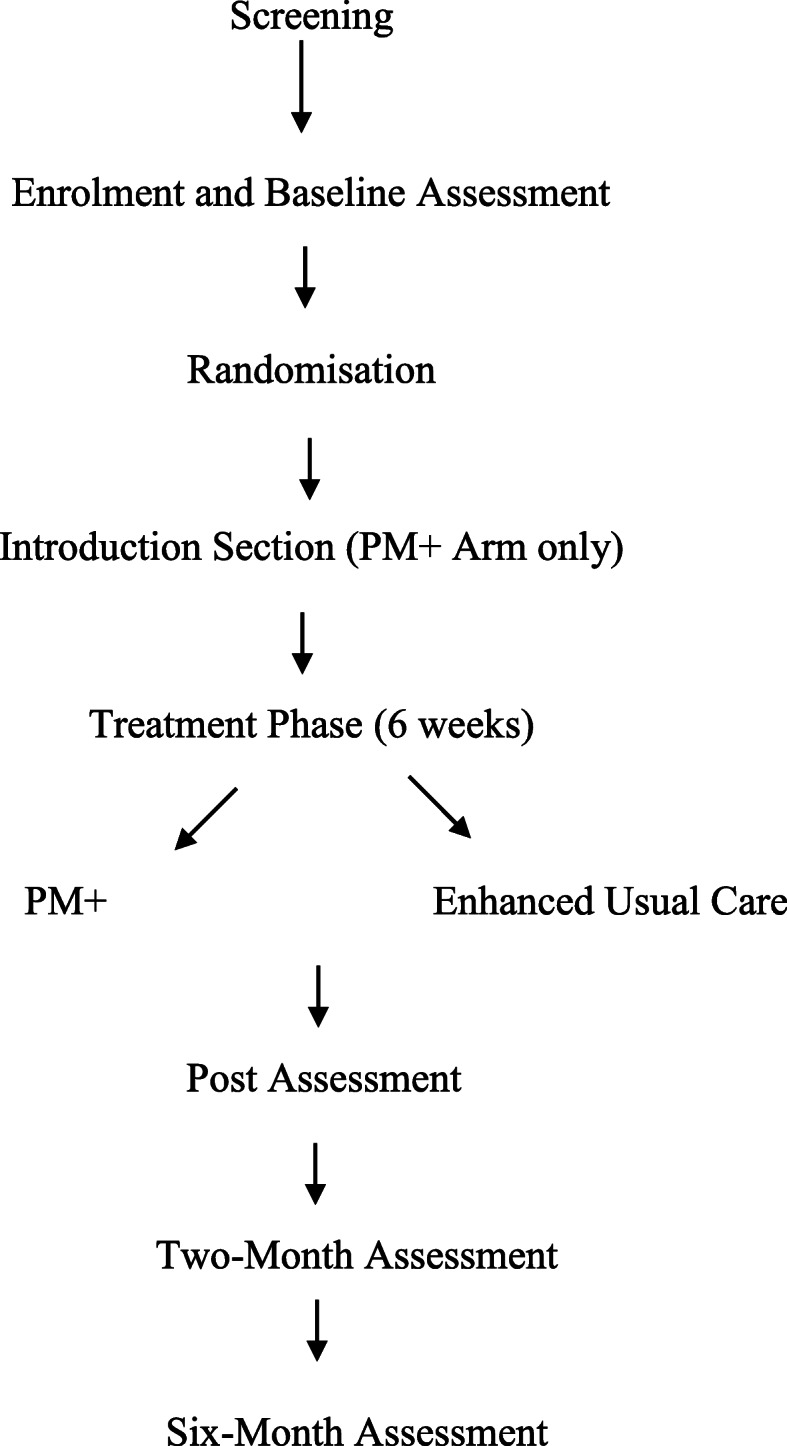
Flow diagram of study

This study aims to evaluate the effectiveness of an adapted version of PM+ to specifically target COVID-19 related distress in reducing anxiety and depression. We will conduct a two-arm single-blind, parallel, randomized controlled trial (RCT) comparing PM+ with enhanced treatment as usual (ETAU) in 240 participants (see Fig. 1 for an overview of the design, following the Standard Protocol Items: Recommendations for Intervention Trials (SPIRIT [14];). This trial adhered to the trial protocol (version 2, April 10, 2020). The study design will employ a 2 (Treatment Condition) × 4 (Assessment Point) factorial design. Participants will be assessed at baseline, post-intervention, and two- and six-month follow-ups. The primary outcome time point is the two-month assessment, and the primary outcome is anxiety and depression. Secondary outcomes include worry, sleep impairment, positive and negative mood, anhedonia, and COVID-19-related concerns.

### Setting

PM+ will be delivered via a video teleconferencing format for community-based people across Australia.

### Participants

articipant inclusion criteria are a) adult (18 years or older); b) score ≥ 3 on the General Health Questionnaire-12; GHQ-12 [15]); and (c) report sufficient English language comprehension. Exclusion criteria are: a) current psychosis; b) imminent suicidal risk; c) current substance dependence (but not abuse); or d) no internet-based access for teleconferencing.

### Recruitment and informed consent

Potential participants will be recruited for the study portal at the UNSW Traumatic Stress Clinic (http://www.traumaticstressclinic.com.au/). The study will be advertised via online media and Facebook. The investigators will make no direct contact with potential participants prior to their approach to the clinic. Individuals who express interest in taking part in the study will read an explanation of the trial, and then provide informed consent by indicating consent via digital endorsement on the trial website.

### Procedure

Following receipt of consent, participants will be directed to a webpage that that will include the General Health Questionnaire (GHQ). If participants score ≥ 3 on the GHQ they will be randomized to either PM+ or ETAU. If participants are not selected because they score below the cut-offs for the GHQ-12, they will be provided feedback on their test outcomes and reasons why they are not eligible for the study will be explained to them. If participants meet any of the exclusion criteria, participants will be referred to seek support from their local physician for further management with specialised services. Participants will then be directed to an online assessment batter, which will include the Hospital Anxiety and Depression Scales (HADS), Generalized Anxiety Disorder Scale (GAD-7), Sleep Impairment Index (SII), and Positive and Negative Affect Schedule (PANAS), Pleasure Scale (PS), Coronavirus Stress Scale (CSS). This online assessment, which will be repeated at post-treatment and 2-month follow-up will ensure independent assessments because no research personnel will administer these. Participants were reimbursed $50 if they completed all assessments.

### Randomization

This randomization be conducted by a computerized software on a 1:1 basis. A research assistant at UNSW who is independent of any other aspects of the treatment study will allocate individuals into the group they are randomized into and invite them for the introductory online session with the group facilitator.

### Introductory online session

Following informed consent, those randomized to the PM+ will be contacted to participate in an initial 10–15 min introductory online session with their group facilitator to explain technological issues involved in using the online teleconferencing platform, and other basic information relating to how the group intervention will operate.

### Interventions

#### Problem management plus (PM+)

The WHO PM+ programme is a brief psychological intervention that seeks to reduce symptoms of stress (e.g. depression, anxiety) for those affected by ongoing adversity. This program was adapted to include evidence-based strategies that specifically target COVID-19 related distress. This includes education about common reactions to COVID-19, stress management, structured problem solving, worry management, behavioural activation and skills to strengthen access to social support. Finally, relapse prevention will involve teaching participants relapse prevention techniques, including identification of possible future relapse situations and rehearsal of strategies to manage these situations. PM+ content was adapted to expand its content to address excessive worries because the nature of the pandemic is that distress can be heightened by worries about health of self and others, employment security, and financial stress, as well adapting the content to manage loss of normal activities during lockdowns and limited access to use social supports. This adapted version of PM+ is delivered over 6 weekly sessions of 60-min duration. In this RCT, PM+ will be delivered in anticipated group size of 3–4 participants via a videoconferencing platform. PM+ group facilitators will be Masters or Doctoral trained clinical psychologists.

Adherence to PM+ protocol will be ensured by weekly group supervisions including the group facilitators and supervisors. To evaluate treatment fidelity, treatment sessions will be audiotaped and 10% of all PM+ sessions will be independently rated for adherence by two independent clinical psychologists. Raters will be required to nominate what therapy components were present in each session and the quality with which these components were administered.

#### Enhanced treatment-as-usual (ETAU)

Participants in the ETAU arm will be emailed a resource package comprising handouts detailing the strategies taught in PM+, with instructions to work through in a self-paced manner across the course of a six-week duration. Those randomized to the ETAU arm will not receive support from a clinical psychologist/group facilitator.

### Screening measure

The General Health Questionnaire (GHQ-12) is a well validated indicator of psychological distress [15] that will be used to screen individuals eligible to partake in the trial (see Table 1). The GHQ-12 comprises of 12 questions about general wellbeing, experience of depressive and anxiety symptoms, and sleep disturbances over “the past few weeks”. Items are scored on a 4-point scale (range 0 to 12; higher scores indicate severe psychological distress), where a cutoff of 3 (using the 4-point, 0–0–1-1, original dichotomous scoring system) will be used to screen individuals with psychological distress in relation to COVID-19 [16].

**Table 1:** Overview of measures administered at assessment time points

Construct	Pre-assessment	Post-treatment	2-months post-treatment^a^	6-months post treatment
Screener				
1. Psychological distress	GHQ-12			
Primary Outcome				
2. Anxiety and depression	HADS	HADS	HADS	HADS
Secondary Outcomes				
3. Generalised anxiety	GAD-7	GAD-7	GAD-7	GAD-7
4. Sleep Impairment	SII	SII	SII	SII
5. Mood	PANAS	PANAS	PANAS	PANAS
6. COVID stress	CSS	CSS	CSS	CSS
7. Anhedonia	Pleasure Scale	Pleasure Scale	Pleasure Scale	Pleasure Scale

Note. ^a^ = Primary outcome time point. *HADS* Hospital Anxiety and Depression Scale, *GAD-7* Generalized Anxiety Disorder-7, *SII* Sleep Impairment Index, *PANAS* Positive and Negative Mood Scale, *CSS* COVID-19 Stress Scale

### Primary outcome

The primary outcome will be severity of anxiety and depressive symptoms as measured using the Hospital Anxiety and Depression Scales (HADS [17]). The HADS is a 14-item scale consisting of two sub-scales: HADS-A (Anxiety, 7 items, range 0–21) and HADS-D (Depression, 7 items, range 0–21). Higher scores indicate more severe anxiety and/or depression. The minimal clinically important difference has been determined at 1.32 for HADS-A and 1.40 for HADS-D [18].

### Secondary outcomes

Worry will be assessed using the Generalized Anxiety Disorder Scale (GAD-7 [19]). This scale has demonstrated good reliability, and validity and is useful in assessing the presence and severity of generalized anxiety, where higher scores indicate more severe symptoms. High levels of sensitivity (89%) and specificity (82%) relative to generalized anxiety disorder have been demonstrated using this scale [20].

Sleep difficulties will be assessed using the Insomnia Severity Index (ISI [21]). The ISI is a 7-item measure of sleep impairment; this study used 5 of the 7 items because of time constraints within a larger clinical interview. The items indexed impairment in sleep onset, maintenance, early waking, disturbance caused by sleep problems, and distress caused by sleep problems (this version will omit items indexing satisfaction with sleep and the extent to which sleep problems were noticeable by others). The internal consistency of the 5-item version is 0.87, and the item-total correlations varied from 0.46 (waking early) to 1.00 (delayed onset sleep), with a mean correlation of 0.57. These values are similar to the values reported for the 7-item measures [22], and validate our use of the 5-item scale. Each item is rated on 5-point rating scale (0–4) providing a potential total score of 28, and the recommended cut-off for identifying insomnia is a score of 12. Accordingly, in recognition that we used 5 of the 7 items, we adopted a pro-rated score of 12 as a conservative index of sleep disturbance.

Mood will be assessed using the Positive and Negative Affect Schedule (PANAS [23]) to provide brief measure of positive- and negative affect (two 10-item mood scales). Respondents are asked to rate the extent to which they have experienced a particular emotion “right now” to capture a present-moment timeframe. Items on this scale broadly capture the affective lexicon and has demonstrated high internal consistency and good convergent and discriminant validity [23].

Anhedonia will be with the Pleasure Scale which asks participants to respond to 36 questions about their capacity to experience pleasure in various situations [24]. Each item is scored on a 5-point scale (range 36–180, with lower scores indicating more severe anhedonia). The scale has strong internal reliability and identifies depressed individuals with extreme anhedonia [24].

COVID-19 related concerns will be assessed using the COVID Stress Scales (CSS) that has been developed to index the extent to which an individual engages in worry about COVID-19 related issues [25]. This scale comprises six domains including (1) fears about the dangers of COVID-19, (2) fears about economic consequences of COVID-19, (3) COVID-19related xenophobia, (4) fear of COVID-19 contamination, (5) traumatic stress symptoms, and (6) excessive checking and reassurance seeking. This was used in the current study to assess specific pandemic related emotional reactions and has evidenced good reliability and internal consistency [25]. Each item is scored on a 5-point scale (range 0–24 on each subscale, with higher scores indicating more severe problems).

### Data management

Adverse events will be monitored at each assessment. Any adverse events that occur during the trial involving untoward medical or psychological occurrence in a participant that is related to the trial will be reported in the study on a standard form and submitted to the study’s Data Monitoring Committee and Trial Sponsor. These adverse reactions will be referred to appropriate services and the relevant psychologist managing this case will follow up at 1 month.

As this is a treatment trial, participants will be required to have their identities known to the study because they are seen on a weekly basis, and then be contacted for follow-up assessment. All data entered by participants during the online surveys collected at pretreatment, posttreament, and follow-up will be entered on an electronic database in a deidentified manner in which participants are allocated a unique identification number. Access will be restricted to research staff directly involved in the study. If the data is moved to any other computer location it will have AES-256 bit encryption. We routinely audio record group therapy sessions in line with CONSORT requirements for subsequent testing of treatment fidelity via independent coding of experts. The research staff working on the project will initially have access to the data via the UNSW Research Long Term Data Store Interface.

### Analysis

A total of 240 participants will be included in the trial. Power calculations were based on previous trials of group-based PM+ [13], and indicated a minimum sample size of 105 participants per group is needed to provide power of 0.95 (alpha = 0.05, two-sided) to achieve an effect size of 0.5 between condition; allowing for attrition of 10% at follow-up, this study aimed to recruit 240 participants. An intent-to-treat analysis using Hierarchical Linear Mixed Modelling will be used to assess differential effects of each condition. This statistical method effectively handles missing data by calculating estimates of trajectories. For the posttreatment analyses between the two conditions, analyses will focus on linear time effects, treatment conditions, and interactions. Fixed effects parameters will be tested with the Wald test (t-test, *p* < .05, two-sided) and 95% confidence intervals. Cohen’s (d) effect size will be calculated for all analyses. The primary outcome measure will be the HADS. The primary outcome timepoint will be the 2 months assessment. Given the speed of this trial, there will be no interim analyses to determine if the trial needs to be terminated prematurely.

## Discussion

Evaluation of an adapted version of PM+ in managing COVID-19 related distress in the Australian population will address the significant and growing mental health needs of this pandemic. Results will be published in peer-reviewed journals, and results will be summarized on the trial website. Specifically, this treatment trial will address the need for accessible, brief, and effective psychological interventions in the face of unpredictable lockdowns, physical distancing and other quarantine measures during this pandemic. If found to be effective, this adapted version of PM+ may be rolled out to other affected settings globally for further adaptation. To this end, this adapted PM+ manual will be published online and made available for rapid use during COVID-19.

## Data Availability

This report is a protocol paper and so no data is involved. Following the trial completion, deidentified data will be available on request.

## Abbreviations

CSS: Coronavirus Stress Scale
ETAU: Enhnaced Treatment as Usual
GAD-7: Generalized Anxiety Disorder Scale
GHQ: General Health Questionnaire
HADS: Hospital Anxiety and Depression Scales
PANAS: Positive and Negative Affect Schedule
PM +: Problem Management Plus
PS: Pleasure Scale
RCT: Randomized Controlled Trial
SII: Sleep Impairment Index
SPIRIT: Standard Protocol Items: Recommendations for Intervention Trials
WHO: World Health Organization
